# *BMC Medical Research Methodology* reviewer acknowledgement 2015

**DOI:** 10.1186/s12874-016-0123-5

**Published:** 2016-02-12

**Authors:** Giulia Mangiameli

**Affiliations:** BioMed Central, Floor 6, 236 Gray’s Inn Road, London, WC1X 8HB UK

## Abstract

The editors of *BMC Medical Research Methodology* would like to thank all our reviewers who have contributed to the journal in Volume 15 (2015).

**Iosief Abraha**

Italy

**Michal Abrahamowicz**

USA

**Nima Aghaeepour**

Canada

**Mussie Akalu**

USA

**Hadi Alizadeh Noughabi**

Iran

**Mohamed Alosh**

USA

**Federico Ambrogi**

Italy

**Pavlina Andreeva-Gateva**

Bulgaria

**Judith Arnetz**

USA

**Jennifer Arney**

USA

**Kristopher Attwood**

USA

**Thomas Augustin**

Germany

**Erin Austin**

USA

**Steven Babbin**

USA

**Aïda Bafeta**

France

**Anna Bagnoli**

UK

**Samprit Banerjee**

USA

**Lola Baydala**

Canada

**Melanie Bell**

USA

**Antonia Bennett**

USA

**Vance Berger**

USA

**Regina Bernal**

Brazil

**Lucie Biard**

France

**Brad Biggerstaff**

USA

**Myriam Blanchin**

France

**Michael Blum**

France

**Amy Bodde**

USA

**Aline Boligon**

Brazil

**Lyndal Bond**

Australia

**Mohamed Boucekine**

France

**Michel Boudreaux**

USA

**James Boyd**

Australia

**Peter Bragge**

Australia

**Christian Brettschneider**

Germany

**Karl Broman**

USA

**Jenni Brooks**

UK

**Hilary Brown**

Canada

**Sylwia Bujkiewicz**

UK

**Peter Burgard**

Germany

**Jonathan Burton**

UK

**Lisa Calderwood**

UK

**Marion Campbell**

UK

**Math Candel**

Netherlands

**Massimiliano Caporin**

Italy

**Brad Carlin**

USA

**Carme Carrion**

Spain

**An-Wen Chan**

Canada

**Yu-Mei Chang**

Taiwan

**Maggie Chen**

Canada

**Bingshu Chen**

Canada

**Xiwei Chen**

USA

**Yuhui Chen**

USA

**Fati Cheraghi**

Iran

**Francesco Chiappelli**

USA

**Jennifer Childs**

USA

**Kacper Chwialkowski**

UK

**Jérémie F. Cohen**

France

**Clare Coleman**

Australia

**Cindy Cooper**

UK

**Shannon Cope**

Canada

**Heather Cordell**

UK

**Enzo Coviello**

Italy

**Katelynn Crick**

Canada

**Joanna Crocker**

UK

**Rada Dagher**

USA

**Dong Dai**

USA

**Eleonora Dal Grande**

Australia

**Sayan Dasgupta**

USA

**Annemarie De Greeff**

UK

**Elizabeth Dean**

USA

**Salome Dell-Kuster**

Switzerland

**Jacques Demotes**

France

**Nandini Dendukuri**

Canada

**Sean Devlin**

USA

**Karla Diazordaz**

UK

**Joseph Dieleman**

USA

**Markus K. Diener**

Germany

**Kai Ding**

USA

**Shanshan Ding**

USA

**Jac Dinnes**

UK

**Sarah Donegan**

UK

**Carola Döpp**

Netherlands

**Vera Ehrenstein**

Denmark

**Deborah Ehrenthal**

USA

**Marinus Eijkemans**

Netherlands

**Andreas Eiset**

Denmark

**Alan Ellis**

USA

**Alexander Engelhardt**

Germany

**Jacques Estève**

France

**Paolo Eusebi**

Italy

**Caroline Fairhurst**

UK

**Hongbin Fang**

USA

**Yixin Fang**

USA

**Debra Fayter**

UK

**Lambert Felix**

UK

**Padhraig Fleming**

UK

**Dean Follmann**

USA

**Maria Joao Forjaz**

Spain

**Ben Francis**

UK

**William Funk**

USA

**Daniel Gaile**

USA

**Paul Gallo**

USA

**Feng Gao**

USA

**Dana Garbarski**

USA

**Emily Geisen**

USA

**Phil Gendall**

New Zealand

**Eveline Geubbels**

Tanzania

**Michael Gionfriddo**

USA

**Jean Golding**

UK

**Manuel Gomes**

UK

**Mithat Gonen**

USA

**Jessica Gorman**

USA

**Erika Graf**

Germany

**Teresa Greco**

Italy

**Mechthild M. Gross**

Germany

**Benjamin Guedj**

France

**Yi Guo**

USA

**Marianne Haapea**

Finland

**Rachael Hageman Blair**

USA

**Alix Hall**

Australia

**Nicholas Hamm**

Netherlands

**Sebastien Haneuse**

USA

**Timothy Hanson**

USA

**Jean-Benoit Hardouin**

France

**James Harrison**

USA

**Markus Hartmann**

Germany

**Ron Hays**

USA

**Sebastian Heidenreich**

UK

**Noor Heim**

Netherlands

**Günter Heimann**

Switzerland

**Daniel Heitjan**

USA

**Enrique Hernández-Lemus**

Mexico

**Ralf-Dieter Hilgers**

Germany

**John Hoey**

Canada

**Rebecca Holman**

Netherlands

**Hwanhee Hong**

USA

**Maria Horne**

UK

**Bo Hu**

USA

**Tania Huedo-Medina**

USA

**Alan Hutson**

USA

**Dan Jackson**

UK

**Martin Jenkins**

UK

**Prabhat Jha**

Canada

**Ming Ji**

USA

**Bin Jia**

China

**Xiaoqian Jiang**

USA

**Hua Jin**

China

**Rebecca Johnson**

UK

**David Johnson**

USA

**Hayley Jones**

UK

**Mona Kanaan**

UK

**Kush Kapur**

USA

**Monika Kastner**

Canada

**Athanassios Katsis**

Greece

**Ada Keding**

UK

**Luke Keele**

USA

**Paul Kelly**

UK

**Friederike Kendel**

Germany

**Antonia Keung**

UK

**Michael King**

UK

**Atle Klovning**

Norway

**Marija Kovandzic**

UK

**Tatsuki Koyama**

USA

**Walter Kremers**

USA

**Oliver Kuss**

Germany

**Samantha Lain**

Australia

**Christophe Lalanne**

France

**George Lambrou**

Greece

**Luís Lapão**

Portugal

**Nicholas Latimer**

UK

**Sebastien Laurent**

France

**Quang Le**

USA

**Minji Lee**

USA

**Sunghee Lee**

USA

**Oliver Lee**

USA

**Boris Lemeshko**

Russian Federation

**Shanshan Li**

USA

**Megan Lim**

Australia

**Wan-Yu Lin**

Taiwan

**Joey Lin**

USA

**Aiyi Liu**

USA

**Shuling Liu**

USA

**Danping Liu**

USA

**Fang Liu**

USA

**Guohui Liu**

USA

**Per Liv**

Sweden

**Bronwyn Long**

Australia

**Mary Losch**

USA

**Thomas Love**

USA

**Tanzy Love**

USA

**Ray Lovett**

Australia

**Qingshu Lu**

Singapore

**Fabio Luciani**

Australia

**Karsten Lunze**

USA

**Sheng Luo**

USA

**Xiaoye Ma**

USA

**Malcolm Macleod**

UK

**Samuel Manda**

South Africa

**Chang Xuan Mao**

China

**Ronald Mataya**

USA

**Gregory Matthews**

USA

**Audrey Mauguen**

France

**Sally Mcclean**

UK

**Geoff Mclachlan**

Australia

**Sanjay Mehendale**

India

**Loren Mell**

USA

**Branko Miladinovic**

USA

**Christopher Miller**

USA

**Joanna Mills Flemming**

Canada

**Kristin Mmari**

USA

**Dylan Molenaar**

Netherlands

**Maureen Monaghan**

USA

**David Morgan**

USA

**Saman Muthukumarana**

Canada

**Laeth Nasir**

USA

**Hannelore Neuhauser**

Germany

**Cattram Nguyen**

Australia

**Pentti Nieminen**

Finland

**Sa-Aat Niwitpong**

Thailand

**Tanja Nordström**

Finland

**Eberechukwu Onukwugha**

USA

**Treena Orchard**

Canada

**Matthew Page**

Australia

**See-Tong Pang**

Taiwan

**Sameer Parpia**

Canada

**Sujata Patil**

USA

**Laure Perrier**

Canada

**Sydney Pettygrove**

USA

**Markus Pfirrmann**

Germany

**Ba' Pham**

Canada

**Siobhan Phillips**

USA

**Dawid Pieper**

Germany

**Melania Pintilie**

Canada

**Romain Pirracchio**

USA

**Maja Pohar Perme**

Slovenia

**Raphael Porcher**

France

**Bernardo Prado**

USA

**Sandip Prasad**

USA

**Elizabeth Quon**

USA

**Daphna Ram**

USA

**Gowri Raman**

USA

**Rasika Rampatige**

Australia

**Marc Ratkovic**

USA

**Daniel Rigby**

UK

**Richard Riley**

UK

**Allysha Robinson**

USA

**Thomas Rusch**

Austria

**Lila Rutten**

USA

**Mike Sachs**

USA

**Mohsen Sadatsafavi**

Canada

**Sandra Safo**

USA

**Rustam Salman**

UK

**Margaret Sampson**

Canada

**Enid Schatz**

USA

**Roberta Scherer**

USA

**Jonathan Schildcrout**

USA

**Björn Schreiweis**

Germany

**Shuan Seaman**

UK

**Véronique Sébille**

France

**Jadbinder Seehra**

UK

**Larissa Shamseer**

Canada

**Meiyu Shen**

USA

**Tsung-Jen Shen**

Taiwan

**James Sheppard**

UK

**Qiuling Shi**

USA

**Yu-Shan Shih**

Taiwan

**Abigail Shoben**

USA

**Haochang Shou**

USA

**Juned Siddique**

USA

**William Siero**

Australia

**Volkert Siersma**

Denmark

**Alla Sikorskii**

USA

**Mark Simmonds**

UK

**Becky Skidmore**

Canada

**Lindsey Smith**

USA

**Janet Smylie**

Canada

**Rebecca Stanley**

Australia

**Alisa Stephens Shields**

USA

**Adrienne Stevens**

Canada

**John Stevenson**

USA

**Ewout Steyerberg**

Netherlands

**Vicky Strauss**

UK

**Lorna Sweeney**

UK

**Yemisi Takwoingi**

UK

**Monica Taljaard**

Canada

**Yuanyuan Tang**

USA

**Johanna Taylor**

UK

**Steven Teerenstra**

Netherlands

**David Torgerson**

UK

**Annette Totten**

USA

**Shaun Treweek**

UK

**Javier Trujillano**

Spain

**Athanasios Tsalatsanis**

USA

**Konstantinos Tsilidis**

Greece

**Ruth Turley**

UK

**Elizabeth Turner**

USA

**Hannu Vähänikkilä**

Finland

**Jan Van De Kassteele**

Netherlands

**Johanna Van Der Lee**

Netherlands

**David Van Klaveren**

Netherlands

**Karen Van Leeuwen**

Netherlands

**Ravi Varadhan**

USA

**Monica Vasquez**

USA

**Dinusha Vatsalan**

Australia

**Craig Velozo**

USA

**Areti Angeliki Veroniki**

Canada

**Wolfgang Viechtbauer**

Netherlands

**Byron Wallace**

USA

**Xiang Wan**

Hong Kong

**Handan Wand**

Australia

**Pengyuan Wang**

USA

**Zhen Wang**

USA

**Yaping Wang**

USA

**James Wason**

UK

**Lesley Weaver**

USA

**David Weiss**

USA

**Rolf Weitkunat**

Switzerland

**Ian R White**

UK

**Sarah White**

UK

**William Whiteley**

UK

**Andreas Wienke**

Germany

**Donald J Willison**

Canada

**Rosemary Wilson**

Canada

**Ed Wilson**

UK

**Julian Wolfson**

USA

**James Wright**

Canada

**Kath Wright**

UK

**Jianrong Wu**

USA

**Vicki Xafis**

Australia

**Fang Xiang**

USA

**Diqiong Xie**

USA

**Tu Xu**

USA

**Jose-Miguel Yamal**

USA

**Wei Yang**

USA

**Hung-Wen Yeh**

USA

**Gui-Shuang Ying**

USA

**Jennifer Yost**

Canada

**Zhiying You**

USA

**Jihnhee Yu**

USA

**Ingeborg Zehbe**

Canada

**Steven Zeliadt**

USA

**Jing Zhang**

USA

**Zhongyang Zhang**

USA

**Daniel Zhao**

USA

**Yize Zhao**

USA

**Jiwei Zhao**

USA

**Yeying Zhu**

Canada

**Jeanette Ziegenfuss**

USA

